# Pediatric Dental Management of an Uncommon Case of Mucopolysaccharidosis Type IV A (Morquio A Syndrome): A Case Report of a Three-Year Follow-Up

**DOI:** 10.1155/2020/2565486

**Published:** 2020-01-22

**Authors:** Andrea Gómez-González, Miguel Ángel Rosales-Berber, Paola De Ávila-Rojas, Amaury Pozos-Guillén, Arturo Garrocho-Rangel

**Affiliations:** Pediatric Dentistry Postgraduate Program, Faculty of Dentistry, San Luis Potosi University, SLP, San Luis Potosi, Mexico

## Abstract

Mucopolysaccharidosis type IV A or Morquio syndrome is an uncommon inherited metabolic condition caused by the deficient intralysosomal storage of glycosaminoglycans. Diagnosis is typically based on clinical examination, skeletal radiographs, and histochemical tests in blood cells or fibroblasts. It is characterized by evident skeletal deformities, poor joint mobility, severe growth deficit, occlusal anomalies, and enamel defects. The aim of the present clinical case report is to describe the general oral management provided to a 6-year-old female patient and its corresponding evolution for more than three years.

## 1. Introduction

Mucopolysaccharidoses (MPS) constitute a group of rare inherited lysosomal storage diseases produced by deficiencies in the metabolism and degradation of special enzymes called glycosaminoglycans (GAGs). These disorders are skeletal dysplasias, in which GAGs are accumulated within lysosomes of skeletal, diverse connective tissues, and teeth, resulting in progressive and permanent skeletal deformities, poor joint mobility, severe growth deficit, coarse facial features, and enlarged organs [1, 2]. MPS are clinically characterized by short stature, mental retardation, multiple dysostoses, cardiovascular/digestive anomalies, ocular impairments, and skin thickening. Early detected mild forms of MPS have a good prognosis and relatively normal life; however, most severe cases exhibit an average survival age of 10 years [3].

The MPS prevalence around the world varies between 1.9 and 4.5 cases per 100,000 live births, depending on the geographic region. MPS have different identified forms, ranging from MPS-I to MPS-VII, according to the clinical/radiographic and biochemical characteristics, the degree of severity, and the involved deficient GAGs [4, 5]. Specifically, the MPS type IV A or Morquio A syndrome (MAS) consists in the intralysosomal accumulation of two GAGs, keratan sulfate, and chondroitin-6-sulfate, interfering with the chondrocyte function in cartilage, bone, and ligaments; this accumulation is due to mutations in the gene encoding of the enzyme N-acetylgalactosamine-6-sulfate sulfatase (GALNS) [6]. MAS is typically featured by the presence of the notorious short neck and trunk, prominent forehead, cervical spinal deformities with cord and medullary compression, general osteoporosis, lungs/heart dysfunction, *pectus carinatum* (malformation of the chest characterized by a protrusion of the sternum and ribs), muscle weakness, kyphoscoliosis, flat feet and knock-knees (or *genua valga*, a condition in which the legs curve inward and the feet are apart when the knees are touching), and frequent falls associated to an abnormal gait [7]. Unlike other MPS, affected children usually exhibit normal intelligence [8]. The most common orofacial anomalies are a short nose and broad mouth; unerupted, malposition, and spaced permanent teeth; abnormally thin and porous enamel and loss of dental structure; anterior open bite associated to macroglossia; and flattened condyle [3, 4, 8–10]. The diagnosis of MAS is carried out through raised urinary levels of keratan sulfate, GALNS activity testing, skin biopsy, leucocyte and fibroblast cultures, genetic analysis, and distinctive radiographic features [8, 11]. General management usually consists of the substitution of abnormal GAGs through enzyme replacement therapies; for more severe cases, the hematopoietic stem cell transplantation is sometimes used [10].

The aim of the present case report is to describe the most representative systemic and orofacial characteristics, diagnostic procedures, and dental management provided to a female pediatric patient aged 6 years and 6 months suffering from Morquio A syndrome. The patient has been followed up closely for more than two years.

## 2. Case Report

In August 2016, a six-year-six-month-old female was referred to the Pediatric Dentistry Postgraduate Program Clinic for routine oral examination and possible treatment. The general and craniofacial features were suggestive of a rare syndrome, with retarded growth according to her chronological age, strange walking, and normal speech. The parents (father 26 and mother 29 years old, both healthy) reported that the patient was diagnosed with a nonsevere form of Morquio syndrome four years ago, confirmed by a significant increase in keratan sulfate in urine and a marked deficiency of galactosamine-6-sulfate activity in leucocyte cultures. No additional diagnostic tests (e.g., genetic analyses) for the condition were undertaken because the condition was diagnosed previously, and they represented a strong monetary outlay for the parents. At that time, the parents were recommended by the medical team to initiate treatment with enzyme replacement; however, the child had not received any type of therapy since the diagnosis by their own decision. The family history did not reveal any other relevant information. According to an exhaustive nutrition/diet questioning through the clinical history, her carbohydrate daily consumption (food and drinks) was considered low.

**Figure 1:**
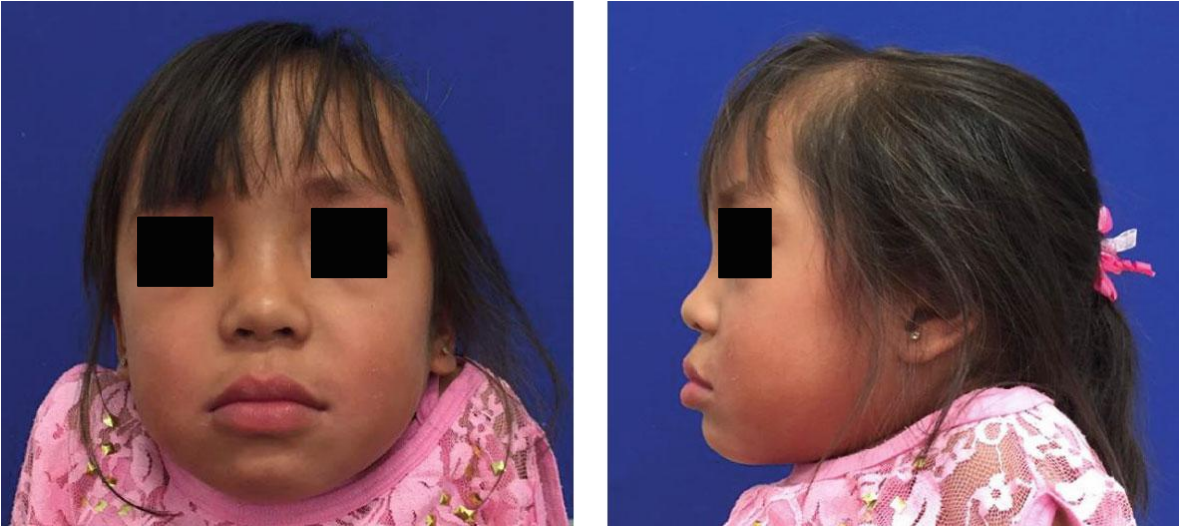
Initial extraoral views (August 2016).

**Figure 2:**
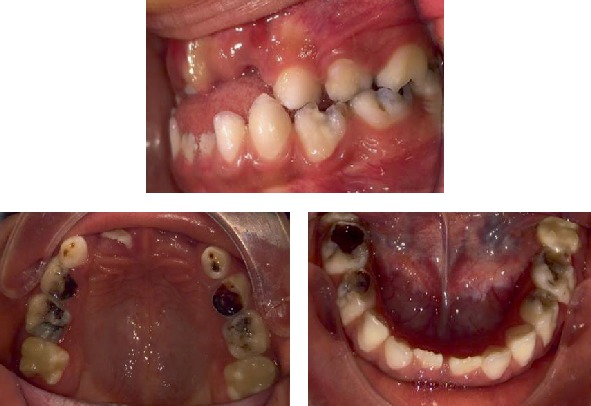
Initial intraoral views (August 2016).

The clinical body examination manifested evident short stature and neck, slight scaphocephaly, bony deformities, protruded chest, stubby hands, kyphosis, and rotated legs. No neurological abnormalities were detected. The most important craniofacial features were brachycephaly, convex/biprotusive profile, flat nasal bridge, closed nasolabial angle, apparent large mouth, and broad lips (Figure 1). Intraoral findings included a partially complete mixed dentition with several deep caries or cavities in all primary molars, ovoid arcades, anterior crossbite, mild open bite, macroglossia, and tongue thrusting; soft tissues were normal, but generalized moderate gingivitis was present; there also was high labial frenum (Figure 2). Parents reported occasional oral abnormal habits such as onychophagia, thumb sucking, and mouth breathing. Both temporomandibular joints were diagnosed as normal, according to a clinical examination involving manual palpation of the masticatory muscles; mouth opening, closing, and lateral movements, or deviations of the mandible; and lateral and dorsal extra-auricular auscultation with the aid of a stethoscope for the determination of abnormal joint sounds. Oral hygiene was considered poor. The patient exhibited significant anxiety and difficulties in maintaining an adequate supine posture on the dental chair.

**Figure 3:**
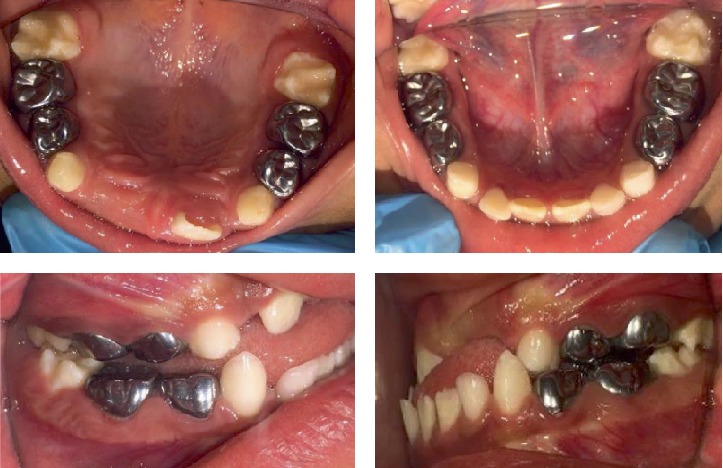
Oral rehabilitation treatment images (October 2016).

**Figure 4:**
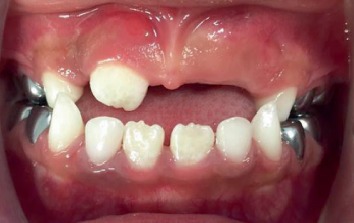
Postrehabilitation treatment (anterior view) (October 2016).

An adequate restorative/endodontic/orthodontic treatment plan was designed and presented to the parents. They agreed and signed an informed consent form. Initially, the patient was behaviorally managed employing persistently different noninvasive psychological techniques (e.g., conditioning, gradual desensitization, “tell-show-do,” and positive reinforcement), dental prophylaxis, and fluoride varnish applications; subsequently, a fair-to-good cooperation level was achieved. The restorative treatment consisted of the placement of preformed metallic crowns on all primary molars. Pulpotomies were performed in the right and left maxillary first and second primary molars, left mandibular first primary molar, and right mandibular second primary molar; and pulpectomies were performed in the left mandibular second primary molar and right mandibular first primary molar, under local anesthesia (lidocaine plus epinephrine) and rubber dam isolation. Pit and fissure sealants were also applied on the four permanent first molars (October 2016) (Figure 3). Additionally, a preventive program was initiated and supervised. Anterior teeth exhibited some small enamel chalky irregularities (Figure 4).

**Figure 5:**
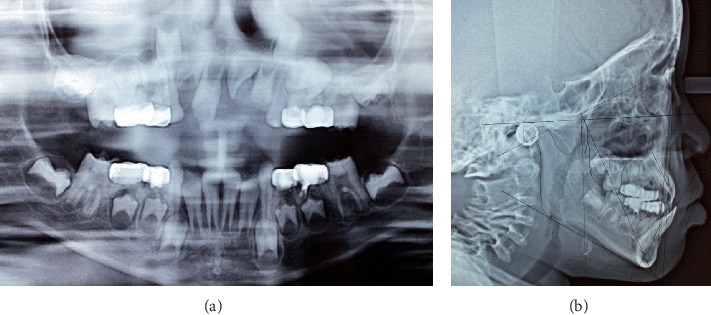
(a) Radiographic view and (b) Ricketts cephalometric analysis (June 2017).

**Figure 6:**
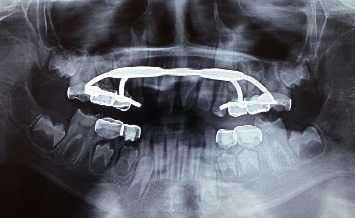
Hyrax appliance (June 2017).

**Figure 7:**
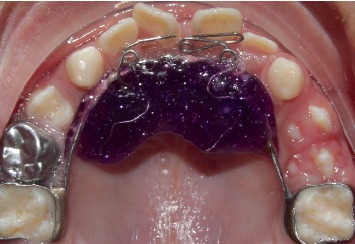
Fixed orthodontic appliance with Z springs (July 2018).

Then, the orthodontic clinical and cephalometric diagnosis (Figures 5(a) and 5(b)) was carried out, determining the presence of a pseudo-class III malocclusion. A Hyrax appliance was placed for four months (June 2017) (Figure 6). In October 2017, Hawley and Schwartz removable orthodontic appliances were placed, and in November 2017, a laser labial frenulectomy was carried out. Nine months later, the Hawley appliance was substituted by a fixed Nance button with “Z” springs over the upper incisors for treating the anterior crossbite (Figure 7).

**Figure 8:**
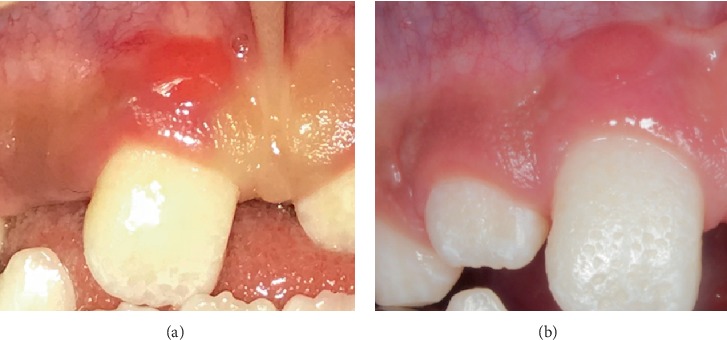
(a) Spongiotic localized juvenile hyperplasia localized above the upper right central permanent incisor (October 2017). (b) The same lesion disappeared without any type of treatment (July 2018).

Unexpectedly, two months later, a gingival lesion appeared above the right upper central incisor, diagnosed presumptively as a “localized juvenile spongiotic gingival hyperplasia (LJSGH)” (Figure 8(a)); after discussing with the oral surgeon, it was decided only to observe the lesion, without any kind of treatment. It healed spontaneously during the next 10 months approximately (Figure 8(b)).

**Figure 9:**
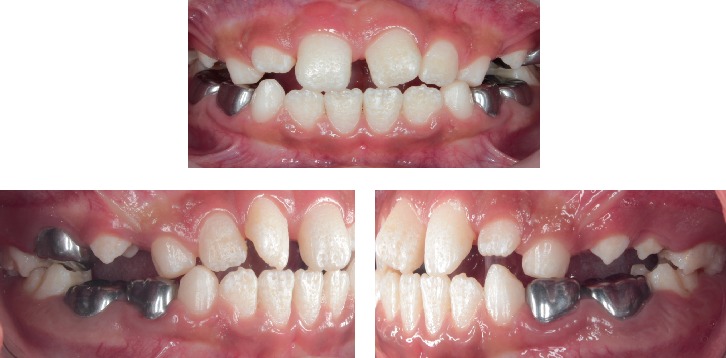
Recent intraoral views (July 2019).

**Figure 10:**
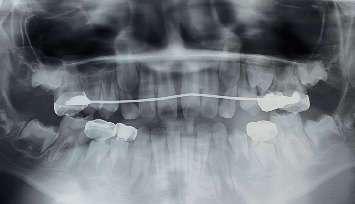
Radiographic control, Nance appliance (September 2019).

In July 2019, the occlusal problems have been almost resolved (Figure 9). The last appointment was in September 2019 for clinical and radiographic control (Figure 10). In this orthodontic stage, the cooperation and participation levels of the child and her parents were considered excellent. The patient will be closely followed in order to assess the evolution of the potential class III malocclusion.

## 3. Discussion

According to the American Academy of Pediatric Dentistry guidelines, one of the most important purposes of this dental area is to provide “both primary and comprehensive preventive and therapeutic oral health care to individuals with Special Health Care Needs (SHCNs),” as an integral part of the pediatric dentistry practice. In this same context, the term SHCNs refers to those children with “any physical, developmental, cognitive, or emotional impairment or limiting condition that requires medical management, healthcare intervention, and/or the use of specialized services or programs” [12]. Mucopolysaccharidosis IV A or MAS is not an exception.

MAS is an uncommon autosomal recessive condition with multisystemic and progressive involvement. The syndrome has two variants: A and B. Type B is caused by a deficiency of *β*-galactosidase, and unlike type A, it usually lacks enamel hypoplasia [10]. Also, the latter finding suggests that GALNS is more determinant for enamel hypoplasia in patients with MAS, who are deficient in the enzyme. In this same regard, these authors theorize that GALNS deficiency may be the result of the abnormal accumulation of chondroitin-6-sulfate within the lysosomes of ameloblasts in the early secretory stage, in the course of odontogenesis [10]. It has been also suggested that during the first phases of amelogenesis, GAGs (mainly keratan sulfate) act as a matrix for a normal attachment between enamel and dentin through the production of amelogenin at the amelodentinal junction. In addition, Al-Jawad et al. [1] demonstrated that, in patients with MAS, deficient keratin sulfate in the dentine tubules disrupts the proper integration of enamel and dentin of primary and permanent teeth and causes an irregular hydroxyapatite crystal orientation and geometry throughout the enamel layer. As a consequence, in MAS-affected children, the occurrence of failures in enamel acid etching is frequent, affecting the adequate bonding of adhesive restorations. These histopathological findings have been as well associated with dental impactions and formation of dentigerous cysts [10]. In addition to the occurrence of enamel hypoplasia, there are other differences between mucopolysaccharidosis IV types A and B. It has been mentioned that patients with type B usually exhibit skeletal dysplasia of the long bones and extremities, with a more normal stature regarding type A; other authors have described the symptoms of type B as being mild (less severe) relative to those observed in patients with MAS [13].

On the other hand, reports along the recent history have mentioned the different oral, dental, and radiographic features in patients suffering from MAS [3, 8, 9, 14–18]. These manifestations are consistent between the studies and are helpful for performing a differential oral diagnosis with other similar disorders, for example, hypoplastic forms of amelogenesis imperfecta (AI), in which dental characteristics notoriously resemble those observed in MAS patients; however, AI lacks skeletal problems [4]. Regarding caries susceptibility, there are contradictory reports in the literature; some authors mention an increased risk for caries development while others establish that the risk is lower [8].

One relevant intraoral sign seen in the present patient was the occurrence of LJSGH during the orthodontic treatment. This is an unusual and painless type of self-limiting gingival inflammatory hyperplasia with female predilection. The lesion is characterized by a micropapillary or granular surface, with a bright red overgrowth on the attached gingiva, which may bleed easily during tooth brushing [19–21]. LJSGH may be clinically confounded with other similar conditions, such as simple inflamed gingiva, puberty gingivitis, pyogenic granuloma, inflammatory gingival hyperplasia, foreign body gingivitis, and linear gingival erythema, among others; demographic and clinical features are sufficient characteristics for an adequate diagnosis [21, 22]. According to Darling and coworkers [23], only persistent lesions should be surgically excised and sent to hystopatological analysis. Lack of response to periodontal therapy or topical corticoid therapy provides additional evidence for the diagnosis of LJSGH [21].

## 4. Conclusions

Pediatric dentistry practitioners must know all relevant bodily, craniofacial, and dental characteristics proper of MAS for early detection and to provide adequate oral management. As mentioned, this disease is frequently confounded with other similar dental conditions, such as AI in its hypoplastic form. Therefore, the pediatric dentist should be part of a multidisciplinary health team in order to perform a precise and opportune diagnosis. Then, it will be possible to offer some advice to the parents regarding the medical management and, of course, on the preventive, endodontic, restorative, and orthodontic procedures for improving the patient's general and oral health and, as consequence, her/his quality of life.
